# Determination of Abundant Metabolite Matrix Adducts Illuminates the Dark Metabolome of MALDI-Mass Spectrometry Imaging Datasets

**DOI:** 10.1021/acs.analchem.0c04720

**Published:** 2021-06-07

**Authors:** Moritz Janda, Brandon K. B. Seah, Dennis Jakob, Janine Beckmann, Benedikt Geier, Manuel Liebeke

**Affiliations:** Max Planck Institute for Marine Microbiology, Celsiusstrasse 1, 28359 Bremen, Germany

## Abstract

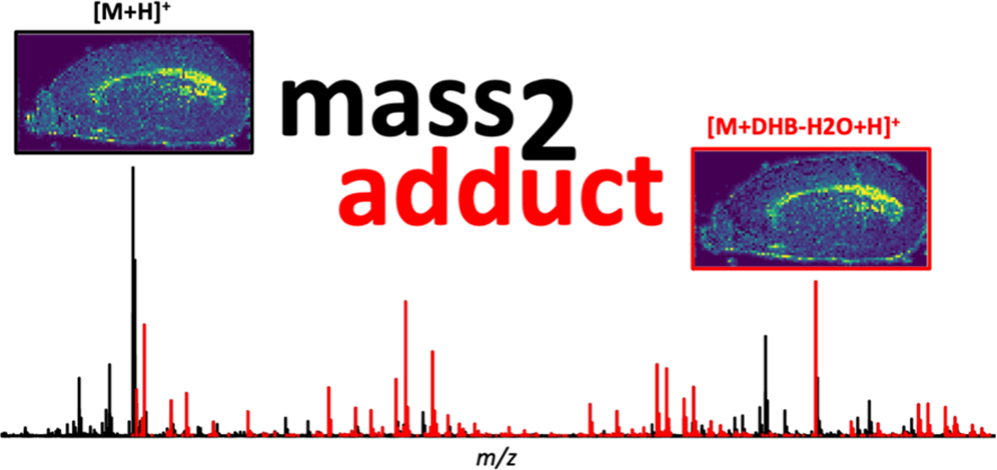

Spatial metabolomics using mass spectrometry imaging (MSI) is a powerful tool to map hundreds to thousands of metabolites in biological systems. One major challenge in MSI is the annotation of *m*/*z* values, which is substantially complicated by background ions introduced throughout the chemicals and equipment used during experimental procedures. Among many factors, the formation of adducts with sodium or potassium ions, or in case of matrix-assisted laser desorption ionization (MALDI)-MSI, the presence of abundant matrix clusters strongly increases total *m*/*z* peak counts. Currently, there is a limitation to identify the chemistry of the many unknown peaks to interpret their biological function. We took advantage of the co-localization of adducts with their parent ions and the accuracy of high mass resolution to estimate adduct abundance in 20 datasets from different vendors of mass spectrometers. Metabolites ranging from lipids to amines and amino acids form matrix adducts with the commonly used 2,5-dihydroxybenzoic acid (DHB) matrix like [M + (DHB-H_2_O) + H]^+^ and [M + DHB + Na]^+^. Current data analyses neglect those matrix adducts and overestimate total metabolite numbers, thereby expanding the number of unidentified peaks. Our study demonstrates that MALDI-MSI data are strongly influenced by adduct formation across different sample types and vendor platforms and reveals a major influence of so far unrecognized metabolite–matrix adducts on total peak counts (up to one third). We developed a software package, *mass*2*adduct*, for the community for an automated putative assignment and quantification of metabolite–matrix adducts enabling users to ultimately focus on the biologically relevant portion of the MSI data.

## Introduction

Mass spectrometry imaging (MSI) techniques enable the visualization of hundreds of metabolites across tissue sections,^1^ biofilms,^2^ and even individual cells.^3^ Spatially resolved metabolomics transforms our understanding of metabolism in biological systems. Identification of all measured molecules is a key challenge in metabolomics, irrespective of the analytical technique used.^4,5^ Mass spectrometry-based metabolomics produces a wealth of data with a very large number of peaks, especially for MSI where one dataset consists of thousands of pixels, each represented by an information-rich mass spectrum. The spectral information is influenced by many factors like the known formation of adducts and less characterized chemical background signals.^5^

Currently, there is a gap in transforming the measured *m*/*z* values to knowledge. This means that we lack easy methods to distinguish chemical background signals from real metabolite signals.^6,7^ One main reason for the lack of signal identification at a molecular level is the structural diversity (isobars and isomers) and dynamic range of metabolites.^8^ In addition, there is a lack of commercial analytical standards (only a few thousands available), which are needed for the identification of a metabolite, according to the metabolite standard initiative.^9^ The typically thousands of measured signals in metabolomics experiments often remain unidentified and have been described as dark metabolome.^10,11^ For untargeted metabolomics approaches using liquid chromatography-mass spectrometry (LC-MS), da Silva et al. estimate that only 1.8% of the spectra can be annotated.^11^ Similarly, low numbers of spectra are expected to be annotated in MSI datasets. In spatial metabolomics, metabolite identifications are further limited by a reduced sensitivity and lower throughput, for instance, during on-tissue fragmentation experiments.

In this study, we focus on metabolite adducts in MSI data and how to determine their presence and abundance. Metabolites typically form adducts with ions derived from inorganic salts or residual water, inherently present in all biological samples. The most common adducts are formed with H^+^, Na^+^, K^+^, or Cl^–^ and are included into mass spectral annotation platforms like Mascot,^12^ METASPACE,^13,14^ and METLIN.^15^ The formation of multiple adducts results in an increased number of signals from one metabolite and simultaneously decreases signal intensity for each individual metabolite adduct peak. The abundance of adducts is influenced by the way the metabolite is ionized (e.g., electrospray or laser desorption) and by the chemical class and concentration of other ions present in the sample. In the case of spatial metabolomics, this effect is a big disadvantage and a source of variability due to changes in local ion concentrations,^16^ directly impacting adduct formation.^17,18^ One of the most commonly used MSI techniques,^19^ MALDI-MSI, additionally uses a highly concentrated matrix layer to aid ionization. MALDI matrices tend to form abundant ion clusters of matrix molecules^20^ and occasionally were described as matrix adducts (e.g., one or more matrix molecules attached to a parent ion).^21,22^ Over the years, these matrix adducts, especially for 2,5-dihydroxybenzoic acid (DHB), have been recognized by the scientific community using MALDI-MS and have been usually discarded as rare “chemical noise”.^5,23,24^ However, the impact of matrix adducts has so far never been quantified in MALDI-MSI datasets from spatial metabolomics experiments.

In this study, we investigated the abundance of adducts across a broad spectrum of MALDI-MSI datasets by leveraging the high mass resolution and the co-localization of matrix–metabolite adducts with their parent metabolites. Our findings with *mass*2*adduct*, developed for adduct abundance estimation, highlight adduct formation of metabolites with known alkali metal ions and also matrix molecules. Our multiplatform comparison across sample types shows that matrix adduct formation is a rather frequent effect during MALDI-MSI. We observed particularly prominent effects when using the most widely applied matrix, DHB, and when employing atmospheric pressure MALDI sources. We propose to include matrix adducts into annotation processes to improve ion identifications in spatial metabolomics datasets to reduce the high percentage of previously unannotated *m*/*z* values, improving the quality of real metabolite annotations.

## Materials and Methods

### MALDI-MSI Datasets

This study is based on 20 MALDI-MSI datasets that were selected with the aim to represent various types of tissue, two different matrices, and three different measurement devices. This included tissue sections of vertebrate brain^25^ and urinary bladder,^26^ marine invertebrates,^27^ marine and terrestrial plants,^28^ and chemical standards. A mixture of 23 chemical standards was spotted on a glass slide and consisted of equal amounts aminomethylphosphonate, carnitine, cellobiose, citric acid, cytidine, dimethylsulfoniopropionic acid, dodecanoic acid, folic acid, glucose, glucose-6-phosphate, glycine, leucine, maleic acid, mannitol, *N*-acetylglucosamine, nonanoic acid, phenylalanine, phosphoglyceric acid, phosphonoacetic acid, pyruvic acid, ribose, thymine, and urea. The data used was acquired on different MSI setups, including three different MALDI sources and three detectors (atmospheric pressure, 337 nm of laser, AP-SMALDI10, Orbitrap (Q Exactive Plus); high vacuum, 337 nm, MALDI2, QTOF (Synapt G2-S), HDMS; high vacuum, 355 nm, SmartBeam-II, MRMS (SolariX)) (for full information, see Tables S2 and S3). MSI datasets referred to as “this study” were acquired with an AP-SMALDI10 setup using an atmospheric pressure matrix-assisted laser desorption/ionization ion source (“AP-SMALDI10”, TransMIT GmbH, Germany), coupled to a Q Exactive HF mass spectrometer (Thermo Fisher Scientific GmbH, Bremen, Germany). MS images were collected with a specified step size (see Table S3) and without overlapping of the laser spots. Mass spectra were acquired in positive-ion mode for all sections prepared with CHCA and DHB using different *m*/*z* ranges (see Table S3) and a constant mass resolving power of 240 000 at *m*/*z* 200.

### Preprocessing of MALDI-MSI Datasets

Peak lists and intensity matrices for datasets #1–3, #5–7, #9, #13–16, #19, and #20 were generated with SCiLS v2019b (Bruker, Germany), using 5 ppm bin width. For full details, see Table S2. Peak intensity matrices were exported in text CSV format. Peak lists were filtered to retain those with an intensity threshold ≥0.05% of the maximum ion intensity of the total ion chromatogram. Datasets #11 and #12 were processed with Waters Imaging software (HDImaging, Waters), retaining the most intense 4000 peaks of the total ion chromatogram. Datasets #4, #8, #10, #17, and #18 were binned with Cardinal MSI v2 at a bin width of 1 mDa, and further processing included a 1% frequency filter (default setting) and a threshold of 0.05% top peak intensity.^29^

### Adduct Identification

Adduct formation increases the ion content and complexity of MALDI-MSI datasets but results in specific mass differences between parent and adduct ions. For each dataset, possible parent–adduct ion pairs were identified in the following way: The mass difference was calculated for all pairs of peaks, and matched against a list of known adduct types^24,30,31^ (see Table S1) within an uncertainty of , where *m*_A_ and *m*_B_ are the parent and adduct masses, respectively, and *p* is the mass accuracy of the processed dataset (see Table S3).

### Controls for Matrix Cluster Ions and False Positives

To identify DHB matrix-only adduct clusters, three MALDI-MSI measurements were performed on slides containing only Super-DHB (9:1 (w/w) mixture of 2,5-DHB and 2-hydroxy-5-methoxybenzoic acid) (Sigma-Aldrich, Steinheim, Germany), with a combined *m*/*z* range of 100–2000. Data were preprocessed in SCiLS as described above with a mass accuracy of 5 ppm and filtered to retain only peaks with intensity threshold ≥0.05% of the maximum intensity peak. For each dataset, peaks matching the combined matrix-only peak list within the uncertainty range (calculated as described above) were subtracted.

For a true matrix adduct, the parent and adduct ions are expected to be positively spatially correlated. Therefore, for each dataset, Pearson’s two-sided correlation test was performed for all ion pairs using per-pixel intensity values. We applied three different methods to screen for false positives: (1) The Bonferroni correction was applied to the p-values from the correlation test, and pairs with corrected *p* ≥ 0.05 were rejected (see all details at https://doi.org/10.5281/zenodo.3363065). (2) Uncorrected *p*-values for positively correlated pairs were used for false-discovery-rate analysis with the R package qvalue v2.10.1,^32,33^ and we applied a *q*-value cutoff of 10^–7^ (see https://doi.org/10.5281/zenodo.3363065). (3) A minimum correlation coefficient cutoff of *r* > 0.3 was applied directly; the first two methods (Bonferroni-corrected *p*-values, *q*-value cutoffs) are effective methods to choose a correlation coefficient cutoff for each dataset (https://doi.org/10.5281/zenodo.3363065).^34^ For the data presented in Figure 3, we chose a correlation cutoff of *r* > 0.1, because it was the most conservative option.

To produce the summary plot, the number of putative adducts above the *q*-value or correlation cutoff for each dataset were tabulated by adduct type as a fraction of the total number of peaks in the mass spectrum and plotted with respect to the instrument platform used to acquire the respective datasets.

For the removal of matrix cluster ions in dataset #1, three MALDI-MSI measurements were acquired with 1024 pixels each of pure Super-DHB, covering the *m*/*z* range 50–2000. Peak lists were created with Cardinal as described above, without the 1% frequency filter (default setting for binning). The peak lists were combined and used as a template from which peaks were subtracted that matched peak list of dataset #1 within a 5 ppm threshold; this removed 2243 matrix peaks from the 8208 original peaks.

The remaining 5965 peaks were reanalyzed for adducts as described above. Identified adduct pair candidates were checked using ion intensity correlations. An intensity matrix for every ion of the respective peak list in each pixel on the dataset was exported with the software MSiReader v0.09.^35^ The intensity matrix was loaded into R, where correlations of adduct pairs were calculated.

### Data and Code Availability

Software to perform the adduct identification and correlation testing are implemented in an R package, *mass*2*adduct*. The software and installation instructions are available on GitHub at https://github.com/kbseah/mass2adduct and are also archived on zenodo (https://doi.org/10.5281/zenodo.1405088). MSI data to replicate the analysis are available at https://www.ebi.ac.uk/metabolights/MTBLS954, and the analysis pipeline and output are archived on zenodo (https://doi.org/10.5281/zenodo.3363065). Data for metabolite standard analysis with DHB and CHCA are available via www.metaspace2020.eu (datasets: MPIMM_221_QE_P_MetaMix, CHCA matrix and MPIMM_222_QE_P_MetaMix, DHB matrix)

### MALDI-MSI Ion Map Processing

MSI ion maps were produced with MSiReader v0.09 using an *m*/*z* tolerance window of ±2.5 ppm and displayed with a modified Jet heatmap without interpolation. Post processing of the exported images such as cropping and resizing was done in Adobe Photoshop CS5.

### Confirmation of Adducts by On-Tissue MS^2^ Measurements

Conformation of selected annotated ions was done via on-tissue MS fragmentation. A consecutive tissue section of dataset #1 was covered with Super-DHB by spraying 30 mg·ml^–1^ Super-DHB in 60:40 acetone/H_2_O (v/v) with 0.1% formic acid onto the sample using the TransMIT matrix sprayer (TransMIT, Gießen). The matrix was sprayed for 30 min with a N_2_ flow of 5 L·min^–1^ and a liquid flow of 7.5 μL·min^–1^. Afterward, the slide was shortly placed into a Petri dish with a drop of methanol for recrystallization.^3^ A 1 Da isolation window and a resolution of 240 000 at *m*/*z* 200 at the mass range 100–900 *m*/*z* were used for MALDI-MS^2^ experiments with the AP-SMALDI10 setup. The sample was manually screened for the presence of target ion with a laser energy of 6.5 μJ. For each mass spectrum, ions of 30 laser pulses were accumulated in the ion trap before they were fragmented with a collision energy of 15 eV via HCD (higher-energy collisional dissociation). A total of 100 spectra were averaged using XCalibur Qual Browser v3.0.63 (Thermo Fisher Scientific, Bremen).

## Results

### Prediction of Abundant Matrix Adducts from the Mass Spectrum

**Figure 1:**
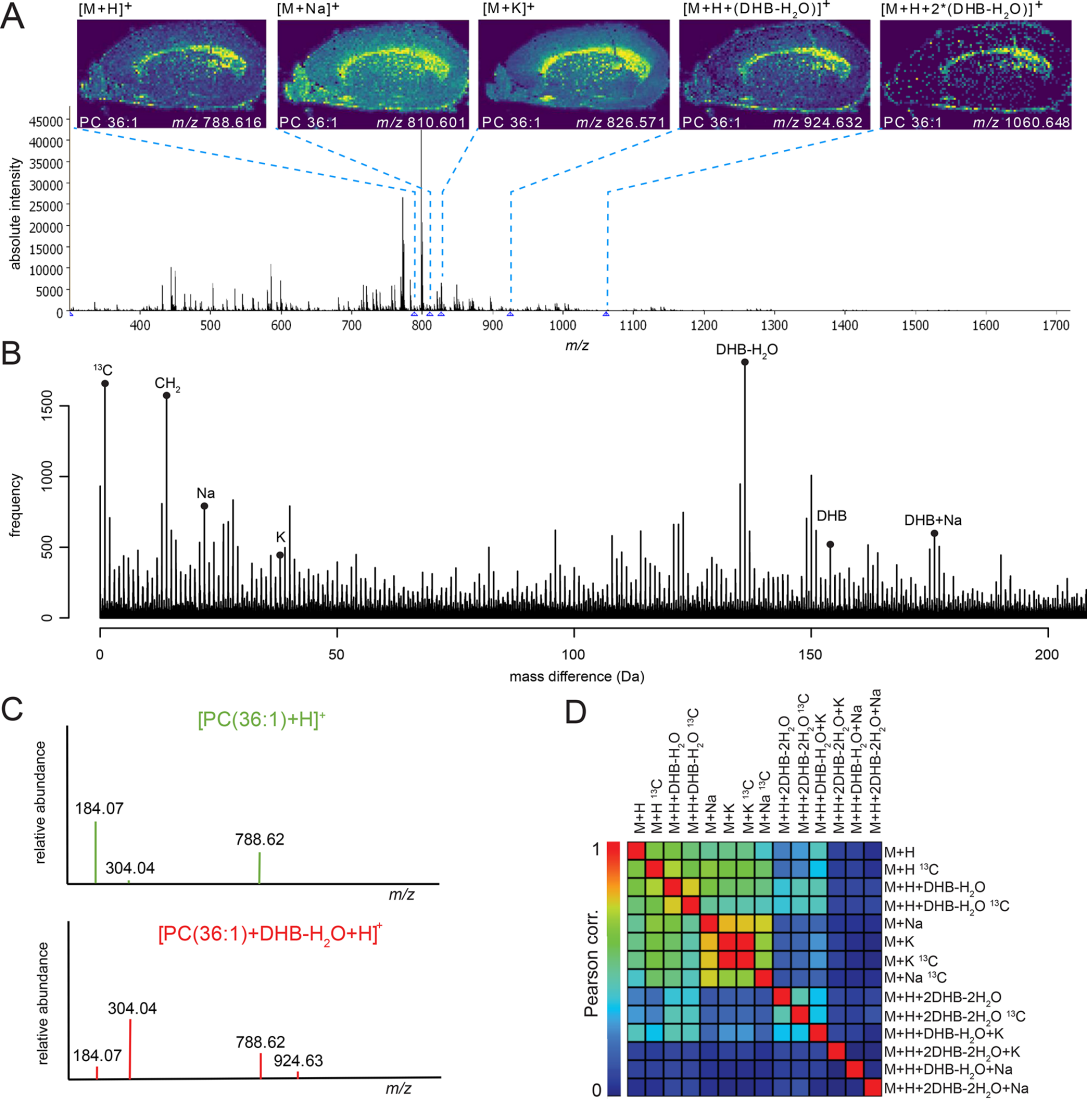
Determination of abundant matrix adducts in MSI data. (A) Total ion spectrum of mouse brain section and spatial metabolite distributions of adducts from PC(36:1) (dataset #10, AP-SMALDI10, DHB matrix). (B) Histogram of mass differences between all peak combinations from mouse brain MSI dataset. (C) On-tissue fragmentation of *m*/*z* 788.616 (top plot) and *m*/*z* 924.632 (bottom plot). (D) Correlation matrix with spatial correlation values (Pearson) of *M* = PC(36:1) and its adducts including natural ^13^C isotope peaks shows a high spatial correlation between [M + H]^+^ and metal adducts as well as matrix adducts.

The types and abundance of matrix adducts in mass spectrometry datasets cannot be predicted a priori. Currently, most studies ignore the chemical background signals and the extent of adduct formation. We aimed to develop a method that enables automatic and unbiased detection of abundant adducts in MSI data. A common method to find adducts is the matching of mass differences between a parent ion and higher *m*/*z* values. By leveraging high mass resolution and high mass accuracy data, ideally below 5 ppm,^36^ the accuracy of detecting specific and common mass difference is higher. We applied this idea to high-mass-resolution MSI data collected in positive ionization mode by first calculating mass differences (Δmass) between all pairs of detected peaks in a MALDI-MSI dataset from a mouse brain tissue section (dataset #10) and then creating a histogram of all Δmass (see Figure 1A). We found high counts of Δmass of 21.985 and 37.955 Da, which matched the Δmass between [M + H]^+^ and [M + Na]^+^ or [M + K]^+^, respectively. This confirmed the presence of abundant Na^+^ and K^+^ adducts, common to animal tissue samples. In addition, the histogram revealed an abundant Δmass of 136.016 Da (2- to 3-fold more counts compared to Na^+^ and K^+^) (see Figure 1A). This Δmass equals C_7_H_4_O_3_ (136.016 Da) and matches the molecular formula of the applied matrix compound DHB without one H_2_O molecule (C_7_H_6_O_4_–H_2_O, 154.027–18.011 Da). The histogram also includes a peak at a Δmass of 154.027 Da, matching DHB. One reason for the high count of Δmass DHB could be the presence of matrix oligomers.^5,24^

### Metabolite Matrix Adduct Confirmation by On-Tissue MS^2^ and Co-localization

To confirm the metabolite–matrix adducts, we performed on-tissue MS^2^ fragmentation experiments on mouse brain tissue section (dataset #10). We chose four pairs of *m*/*z* values with Δmass = 136.016 Da and performed on-tissue MS^2^ on each (Figures 1B, S1, and S2). One example of a parent ion that matched the molecular formula of a membrane lipid was phosphocholine (PC(36:1), molecular formula C_44_H_86_NO_8_P), detected as [M + H]^+^ at *m*/*z* 788.617. [PC(36:1) + H]^+^ showed identical fragments in the MS^2^ spectra as *m*/*z* 924.632 (Figure 1B). We identified *m*/*z* 924.632 as [M + (DHB-H_2_O) + H]^+^ adduct of PC(36:1), including the fragment *m*/*z* 788.616 as neutral loss of DHB-H_2_O (Figure 1B). Further, the similar spatial distributions (corr. coeff. *r* = 0.9, Pearson) supported the hypothesis of the formation of the metabolite–matrix adduct pair between [M + H]^+^ and [M + (DHB-H_2_O) + H]^+^ (Figures 1C and S3). Focusing on PC(36:1) revealed a multitude of adducts (15 in total) including nine metabolite–matrix adducts (see Figure 1D). We found that the neutral addition of matrix molecules is not restricted to the [M + H]^+^ ion but also occurs with metabolite-alkali adducts (e.g., [M + Na]^+^). Our analysis indicated a second matrix adduct to PC(36:1) [M + 2(DHB-H_2_O) + H]^+^ (*m*/*z* 1060.648, corr. coeff. *r* = 0.324) and a third one [M + 3*(DHB-H_2_O) + H]^+^ (*m*/*z* 1196.667, corr. coeff. *r* = 0.063) to the [M + H]^+^ of PC(36:1). We show that DHB does not only form clusters as reported previously,^37^ but results in additional metabolite adducts. Similarly to the Na^+^ and K^+^ adducts, DHB adduct cluster peaks spatially correlate with the [M + H]^+^-ion distribution (see Figure 1D). In summary, nearly 50% of detected adduct types from PC(36:1) are DHB-derived adducts, thus heavily influencing the mass spectral content of MSI data.

### Single Metabolites Can Form a Multitude of Adducts

**Figure 2:**
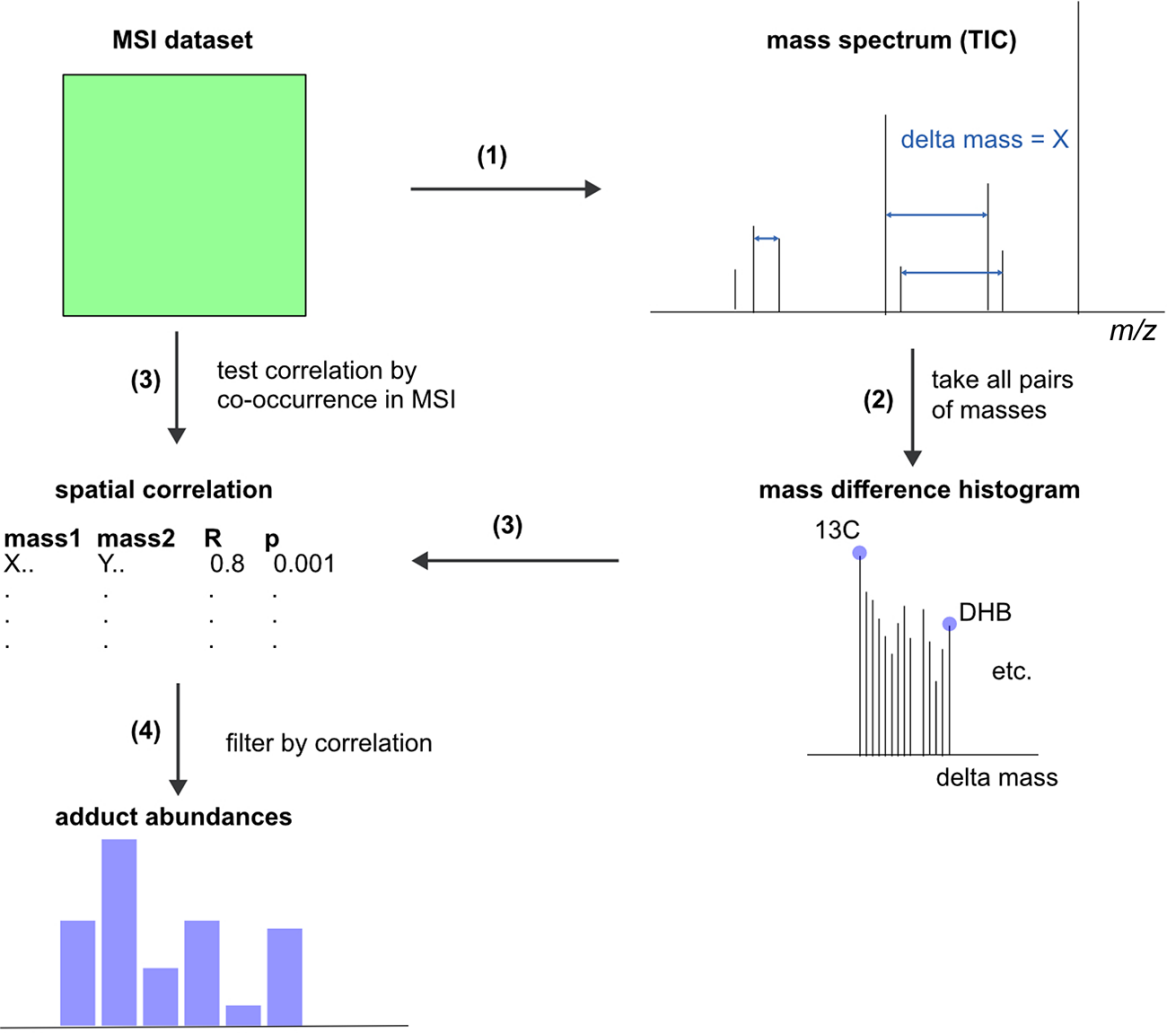
Adduct counting and spatial correlation with *mass*2*adduct*. MSI data in imzML format or intensity matrices in CSV format can be imported to *mass*2*adduct*. (1) *mass*2*adduct* takes all possible pairwise masses and calculates the mass difference for each pair. (2) A mass difference histogram is matched to a list of known mass differences to identify adducts. (3) Spatial correlation of *m*/*z* values is used to identify co-occurring metabolites. Then, a Pearson correlation coefficient is used to validate identified adducts. (4) Filtering with a correlation cutoff excludes false positives from identified adducts.

The fact that one metabolite can form multiple adducts with the matrix and other metal ions prompted us to build a workflow for automated screening for adducts. Our approach is based on two major steps, calculating Δmass values between all peaks of a dataset and testing for spatial correlation only between parent and putative adduct ions (see Figure 2). We compiled a set of matrix-related adducts into a list of adducts and mass differences for typical chemical transformations^30,38^ to match calculated Δmass values of a dataset (see Supporting Table S1).^30^ We used the list to identify the number of possible adducts based on specific *m*/*z* differences for each ion pair in a dataset. Our workflow is available as a software package for R, called *mass*2*adduct*. It allows data import in various formats (e.g., imzML via Cardinal,^29^ intensity tables in CSV format), parallelization to speed up correlation testing, and several visualization tools. The input data needs to be generated with high mass accuracy (below 5 ppm) to allow for an accurate and reliable detection of Δmass for ion pairs. Detection of peaks in each single mass spectrum of an MSI dataset is a challenging task, and rigorous care should be taken to perform this step as accurate as possible. One important measure to consider is the signal-to-noise ratio and its estimation using, e.g., three-sigma rule across all spectra of a dataset. Signal intensity thresholds for each dataset should be adopted based on the data, including testing whether the data is normal distributed.^39^

### Spatial Correlation Analysis of Metabolite Adducts Reduces False-Positive Hits

The experimental confirmation of every potential adduct identified by the *mass*2*adduct* analysis with on-tissue MS^2^ measurements would not be feasible with current methods, especially for multiple datasets. Therefore, we propose a fast and universally applicable approach to screen for potential adduct annotations via spatial correlation analysis.^40^ Adducts are expected to co-occur in the same spot as their parent metabolite for any given tissue sample. Consequently, calculating individual correlation values between the intensities of a peak and its putative adduct peak across all pixels of a MALDI-MSI dataset provides statistical support for MS^1^-based adduct identifications (see spatial correlations between possible adducts of PC(36:1), Figure 1D).

We showcase the identification of adducts using the distribution, identity, and co-localization of the lipid PC(36:1) and its adducts (Figure 1D). The parent ion [PC(36:1) + H]^+^ showed a similarly strong ion intensity correlation to its matrix adduct [PC(36:1) + (DHB-H_2_O) + H]^+^ (corr. coeff. *r* = 0.779) and to its respective ^13^C isotope peak [PC(36:1)_13C_ + H]^+^ (corr. coeff. *r* = 0.883) (Figure 1D). A positive correlation value alone does not guarantee that two ions are chemically related. However, in combination with our prior knowledge on sample and matrix composition, it provides additional confidence to screen for potential adducts. Although changes in local sodium or potassium concentrations can impede strong correlations,^16^ an effect similar to strong local ion suppression occurs. The correlation values can be influenced by very low abundant ion pairs^41^ or the presence of isobaric metabolites with overlapping monoisotopic distribution patterns.

We extended our correlation analysis to every proposed adduct pair within a dataset, which enabled us to remove false positives. Our final *mass*2*adduct* approach provides estimates on the adduct composition of a dataset through (1) adduct counting by Δmass calculation, (2) excluding matrix cluster ions (see the Materials and Methods section) from the list of ion pairs, and (3) excluding nonspatially related ions by performing a correlation test and false-discovery-rate (FDR)-based analysis of the remaining adduct pairs (see Figure 2). We included an output analysis into *mass*2*adduct*, highlighting the peaks of a total ion count spectrum that are related to DHB adducts (see Figure S5B)

### Metabolite Adduct Composition across Multiple Samples and MALDI-MSI Systems

We extended our *mass*2*adduct* analysis toward a broad spectrum of MSI datasets, covering tissue sections of vertebrate brain^25^ and urinary bladder,^26^ marine invertebrates,^27^ marine and terrestrial plants,^28^ and chemical standards. The data was acquired on different MSI setups, including three different MALDI sources and detectors (for a full list of datasets and settings, see Tables S2 and S3).

**Figure 3:**
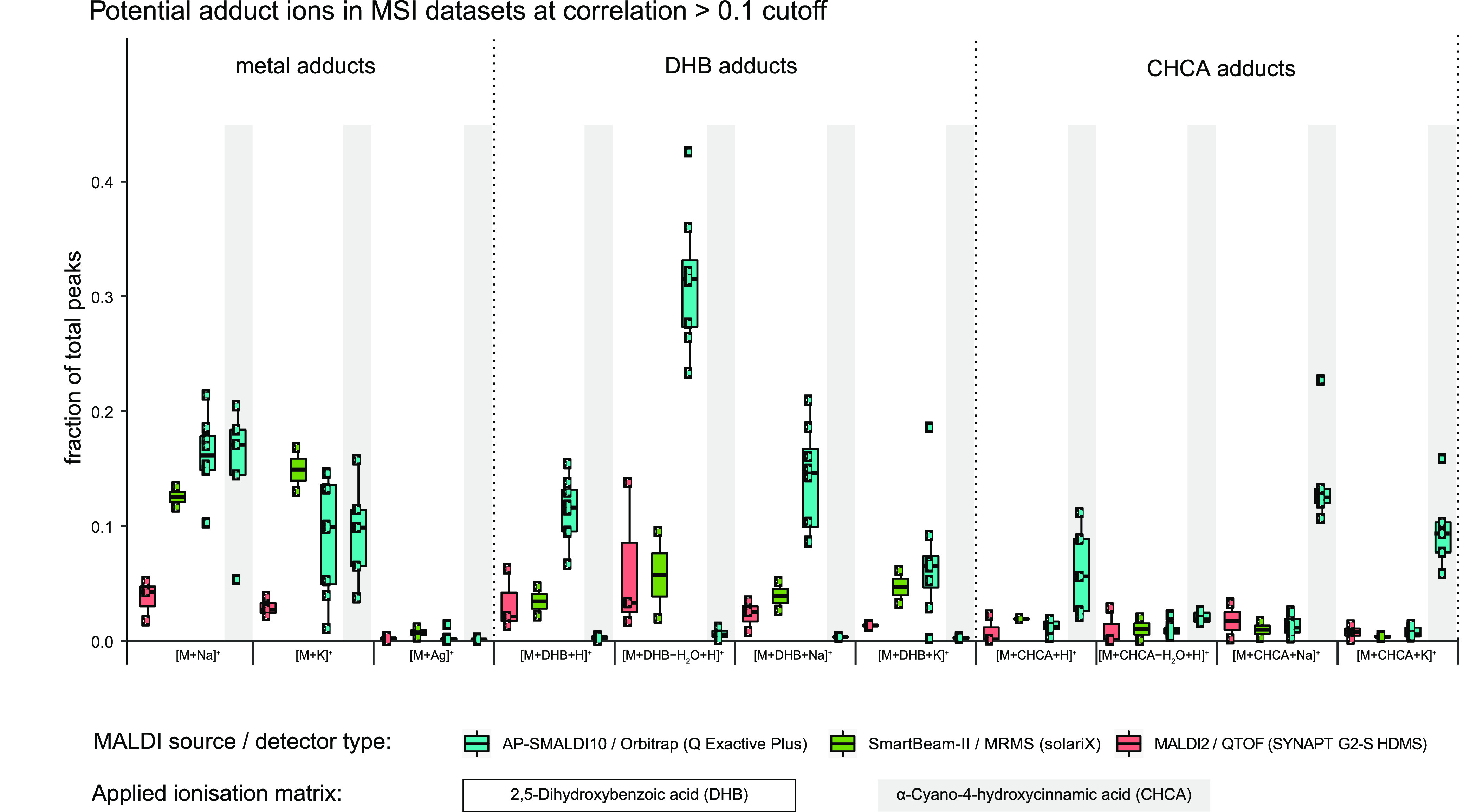
Adduct counts across 16 MSI datasets from four instrument systems. For each MSI system (AP-SMALDI10/Orbitrap (Q Exactive Plus); SmartBeam-II/MRMS (SolariX); MALDI2/QTOF (SYNAPT G2-S HDMS)), the fraction of total peaks from multiple datasets is shown (AP-SMALDI10: DHB *n* = 7, CHCA *n* = 5; SmartBeam-II: DHB *n* = 2; MALDI2 DHB *n* = 3; DHB matrix: white background; CHCA matrix: gray background). To exclude false positives, a cutoff correlation coefficient >0.1 was applied. Using a false-discovery rate cutoff <10^–7^ produced qualitatively similar results (see https://doi.org/10.5281/zenodo.3363065).

With *mass*2*adduct*, we detected comparable Na^+^ and K^+^ adduct counts across different tissue types and MALDI systems. Notably, we found abundant [M + (DHB-H_2_O) + H]^+^ adducts in all datasets prepared with the DHB matrix. We tested if our approach could detect adducts from other matrices and included samples prepared with α-cyano-hydroxy-cinnamic acid (CHCA), another commonly applied MALDI-MSI matrix. Compared to DHB, CHCA formed fewer matrix adducts based on the counts of peaks with Δmass = 189.04 Da (Figure 3). The fractions of total peaks matching ^13^C isotope (Δmass = 1.003) mass difference were amongst the highest and did not show a large variance between different matrices and MSI setups. In our comparison, atmospheric pressure MALDI-MSI (AP-SMALDI10/Orbitrap) showed the highest abundances of [M + (DHB-H_2_O) + H]^+^ adducts with ∼31% of all peaks (equals 0.31 fraction of peaks, see Figure 3) compared to high-vacuum MALDI-MSI datasets (e.g., SmartBeam-II/MRMS ∼6%; MALDI2/QTOF ∼6%). This is in agreement with earlier observations of the adduct composition varying between atmospheric and high-vacuum MS systems.^21^

In MSI datasets treated with CHCA matrix, [M + CHCA + Na]^+^ with Δmass = 212.032 Da was found to be the major matrix adduct with ∼0.1 fraction of peaks (see histogram of datasets #5, #6, #7, and #18, https://doi.org/10.5281/zenodo.3363065; see fraction of peaks in Figure 3, gray background). However, CHCA datasets included also ion pairs whose mass difference matched [M + (DHB-H_2_O) + H]^+^ adducts (counts 0–1.2% of total peaks). Such false-positive matches possibly originate from metabolites, different in their molecular formula by C_7_H_4_O_3_. The same applies for Δmass of CHCA adducts in datasets prepared with DHB as matrix (fraction of peaks Δmass CHCA <1% for “AP-SMALDI/Orbitrap,” <2% for “SmartBeam-II/MRMS”). This prompted us to include an implausible adduct like silver (Ag^+^), similar to an approach for FDR-controlled metabolite annotation.^14^ Ag is a rare noble metal that has no known biological function and is therefore highly unlikely to be present in relevant concentrations in tissue samples. Δmass = 105.897 Da for ^107^Ag^+^ was detected in every dataset at very low abundances (counts of approximately 0.001 fraction of peaks). This detection rate of <1% is much lower compared to the ∼1% DHB or CHCA adduct counts in datasets which were not prepared with the respective matrix. This may be explained with the fact that DHB and CHCA are organic C_x_H_y_O_z_ compounds and well resemble naturally occurring differences between molecules. Although Ag is an inorganic compound with a unique mass defect, it is unlikely to match Δmass values in tissue samples.

### DHB Adducts Can Cause False-Positive Metabolite Annotations

To determine which metabolites form adducts with DHB, we compared the *mass*2*adduct* peak list toward results of an MSI metabolite annotation platform (www.metaspace2020.eu).^13,14^ An example dataset from invertebrate tissue (dataset #1) showed that out of 604 possible *m*/*z* values with matching peaks to DHB adducts, 103 of the “parental” ions have been annotated as metabolites (dataset: MPIMM_030_QE_P_BP_CF_10, FDR <20%, annotation database: LIPID_MAPS-2016). Almost all annotated *m*/*z* values were lipids from different groups, mainly phosphocholines (*n* = 62), phosphoethanolamines (*n* = 37), and sphingolipids (*n* = 12). Among this list of ion pairs, one case was found with *m*/*z* 742.575 [M + H]^+^ of monounsaturated PC lipid (PC(P-16:0/18:2), molecular formula C_42_H_80_NO_7_P) and its annotated DHB adduct [M + (DHB-H_2_O) + H]^+^*m*/*z* 878.591. The annotation was polyunsaturated phosphatidylserine (PS) lipid (PS(21:0/22:6), molecular formula C_49_H_84_NO_10_P), with a difference of C_7_H_4_O_3_ to (PC(P-16:0/18:2)). This example highlights that DHB adducts can be the cause for false-positive annotations if only the exact mass is used for annotation. Further MS^2^-based on-tissue identification will be needed to verify that such correlation pairs are DHB adducts and not based on the co-localization of two endogenous compounds with C_7_H_4_O_3_ difference.

### Small Polar Nonlipid Metabolites Form Matrix Adducts

With MALDI-MS research focusing on lipids,^23^ lipopolysaccharides, and proteins,^42^ the formation of metabolite–matrix adducts was initially noticed for larger molecules only (500–1200 Da).^5,24^ Our investigation of MSI datasets measured in a small mass range (50–500 Da) revealed metabolite matrix adducts for small metabolites. We processed mussel tissue and spotted chemical standards analyzed with the AP-SMALDI setup and the matrices DHB (datasets #4 and #17) and CHCA (datasets #8 and #18). We identified a comparable fraction of total peaks as matrix adducts, shown in Figure 3 (e.g., fraction of total peaks for [M + (DHB-H_2_O) + H]^+^ = 0.36 (#4) and 0.32 (#17)).

**Figure 4:**
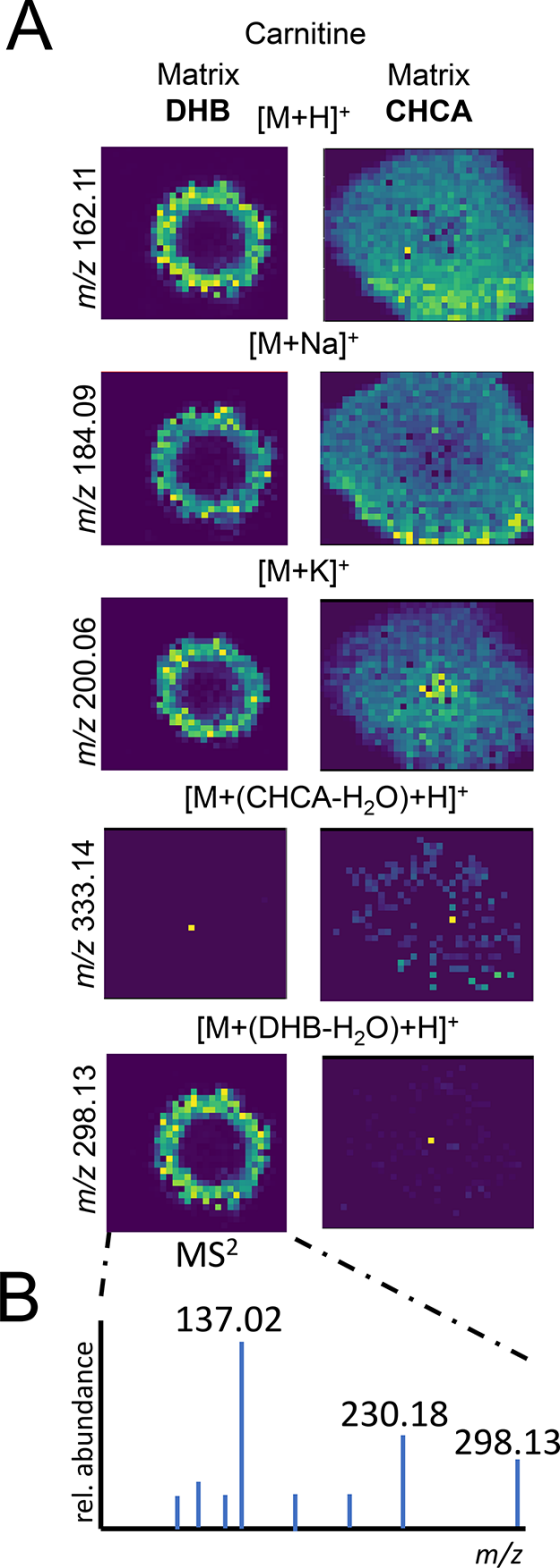
Carnitine forms adducts with DHB and CHCA. (A) Ion maps of carnitine and its adducts, standard spotted and analyzed with DHB (left) and CHCA (right) as matrix. (B) MS^2^ spectrum of carnitine adduct with DHB [C_7_H_15_NO_3_ + (DHB-H_2_O) + H]^+^ (*m*/*z* 298.13) fragmented with normalized collision energy 35; the most abundant fragment 137.02 matches the mass of DHB-H_2_O ([C_7_H_4_O_3_ + H]^+^ calc. 137.02 Da).

A mixture of 23 pure chemical standards contained amino acids, sugars, fatty acids, and other organic acids (see the Material and Methods section) was analyzed using DHB as matrix in positive ionization mode. We detected 16 out of 23 standards, of which 7 showed at least one metabolite matrix adduct. All metabolites with a matrix adduct contained at least one amine group in their structure (i.e., aminomethylphosphonate, carnitine, cytidine, folic acid, leucine, *N*-acetylglucosamine, phenylalanine), whereas metabolites without nitrogen showed no matrix adducts (e.g., sugars, small organic acids) (see Figure 4A). We could confirm the carnitine-DHB adduct via MS^2^ (Figure 4B). The same metabolite mixture analyzed with CHCA showed metabolite–CHCA adducts as well, but less abundant compared to DHB adducts. Our results show that metabolite–matrix adducts occur not only with lipids (e.g., amine-containing phosphocholines and sphingomyelins^23,24^) but also with amino acids and other amine-group-containing metabolites. Whether this effect is transferable to negative-mode ionization needs to be determined.

## Conclusions

In summary, our study shows that metabolite–matrix adducts, previously considered to be negligible chemical background signals, can be abundant across major MSI systems and sample types. This poses an issue for peak identifications if not considered. We developed a software pipeline *mass*2*adduct* to perform a simple mass difference calculation and spatial correlation analysis as a rapid and efficient way to screen for these adducts in existing MSI datasets. Considering the thousands of MALDI-MSI datasets measured (e.g., available at www.metaspace2020.eu(13)), each containing thousands of detected signals, it is crucial to acknowledge the high frequency of matrix adducts for identifications. Our results suggest that including metabolite–matrix adducts into database annotations can reduce the number of unannotated peaks and, on the other hand, will prevent possible false annotations and biological misinterpretations. Our findings also show the need for MSI-independent verification of annotated metabolites using an orthogonal method. Suitable approaches could be microsampling or laser capture microdissection with following LC-MS analysis.^43^ Taken together, our results highlight that the dark metabolome of MALDI-MSI datasets might be not so dark after all, but merely clouded by the signals added through matrix adducts.

## Supporting Information Available

On-tissue MS^2^ mass spectra and respective MS images of parent ions and DHB matrix adducts from mouse brain dataset #10; ion maps for PC(36:1) (mouse brain dataset #10) and respective adduct ion maps; mass2adduct workflow in R; output options from mass2adduct; list of mass differences of adducts and chemical transformations; list with details about analyzed samples; and list with details about analyzed MSI datasets (PDF)
