# Supplementary material for: Determination of Abundant Metabolite Matrix Adducts Illuminates the Dark Metabolome of MALDI-Mass Spectrometry Imaging Datasets
Source: Anal Chem. 2021 Jun 7;93(24):8399–407. doi: 10.1021/acs.analchem.0c04720 (PMC8223199; doi:10.1021/acs.analchem.0c04720)
Supplement: Supplementary file 1 — ac0c04720_si_001.pdf [file ac0c04720_si_001.pdf]

Supporting information

Determination of abundant metabolite-matrix adducts illuminates the dark metabolome of MALDI-mass-spectrometry imaging datasets

*Moritz Janda[1§], Brandon K. B. Seah[1,2§], Dennis Jakob[1], Janine Beckmann[1], Benedikt Geier[1], Manuel Liebeke[1]**

[1]Max Planck Institute for Marine Microbiology, Celsiusstrasse 1, 28359 Bremen, Germany

[2]Max Planck Institute for Developmental Biology, Max-Planck-Ring 5, 72074 Tübingen, Germany

Contents:

**Figure S 1:** MS^2 mass spectra and respective MS images from mouse brain dataset #10	S-2
**Figure S 2:** MS^2 mass spectra and respective MS images from mouse brain dataset #10	S-3
**Figure S 3:** Ion maps for PC(36:1) and respective adducts from mouse brain dataset #10	S-4
**Figure S 4:** *mass2adduct* workflow in R	S-5
**Figure S 5:** Output options from *mass2adduct*	S-6
**Table S 1:** List of mass differences	S-7
**Table S 2:** Sample metadata	S-10
**Table S 3:** Mass spectrometry metadata	S-11

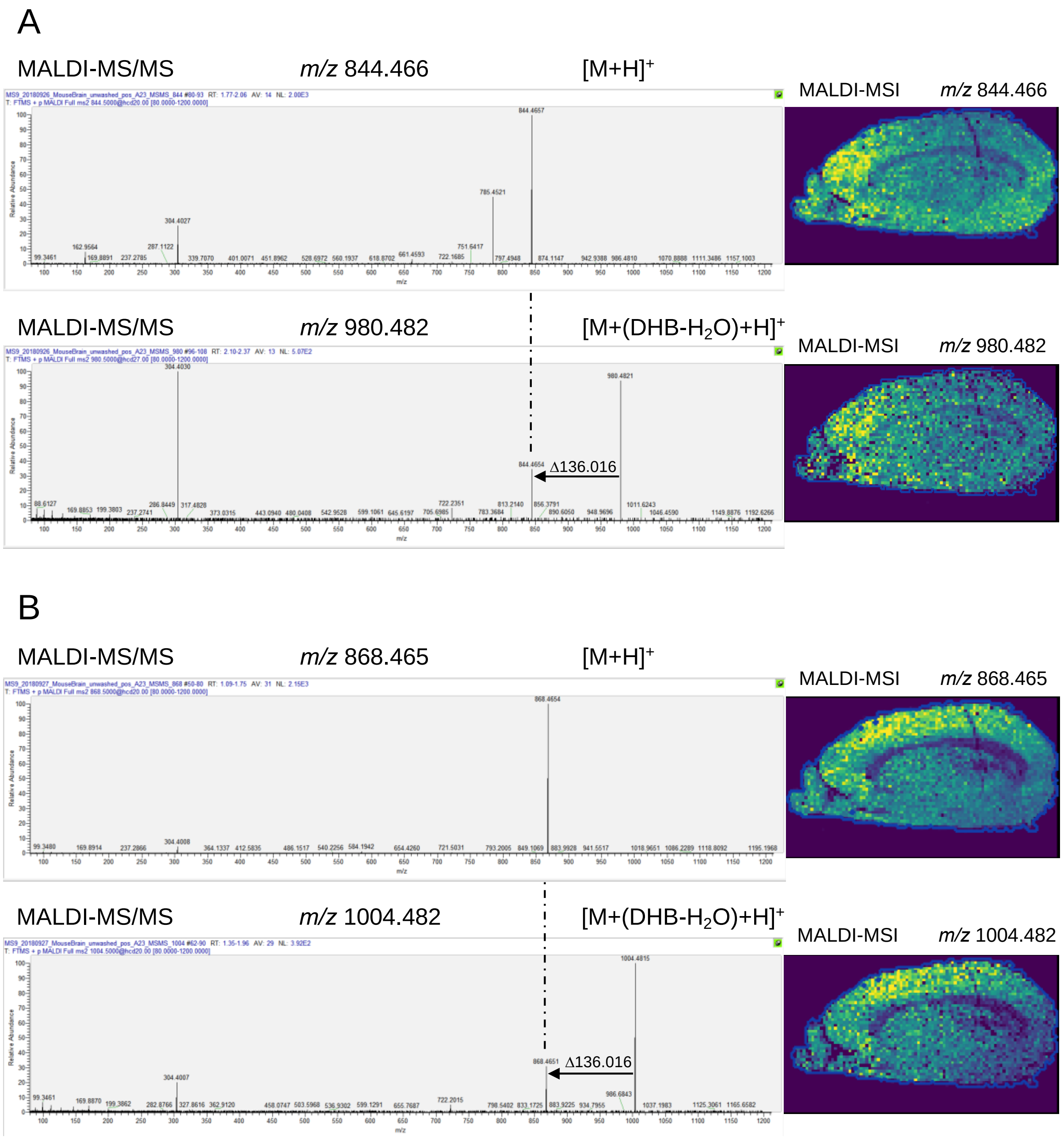

Figure S 1: MS^2 mass spectra and respective MS images of parent ions and DHB matrix adducts from mouse brain dataset #10. **A)** $[M+H]^+$ = *m/z* 844.466, $[M+(DHB-H_2O)+H]^+$ = *m/z* 980.482 **B)** $[M+H]^+$ = *m/z* 868.465, $[M+(DHB-H_2O)+H]^+$ = *m/z* 1004.482

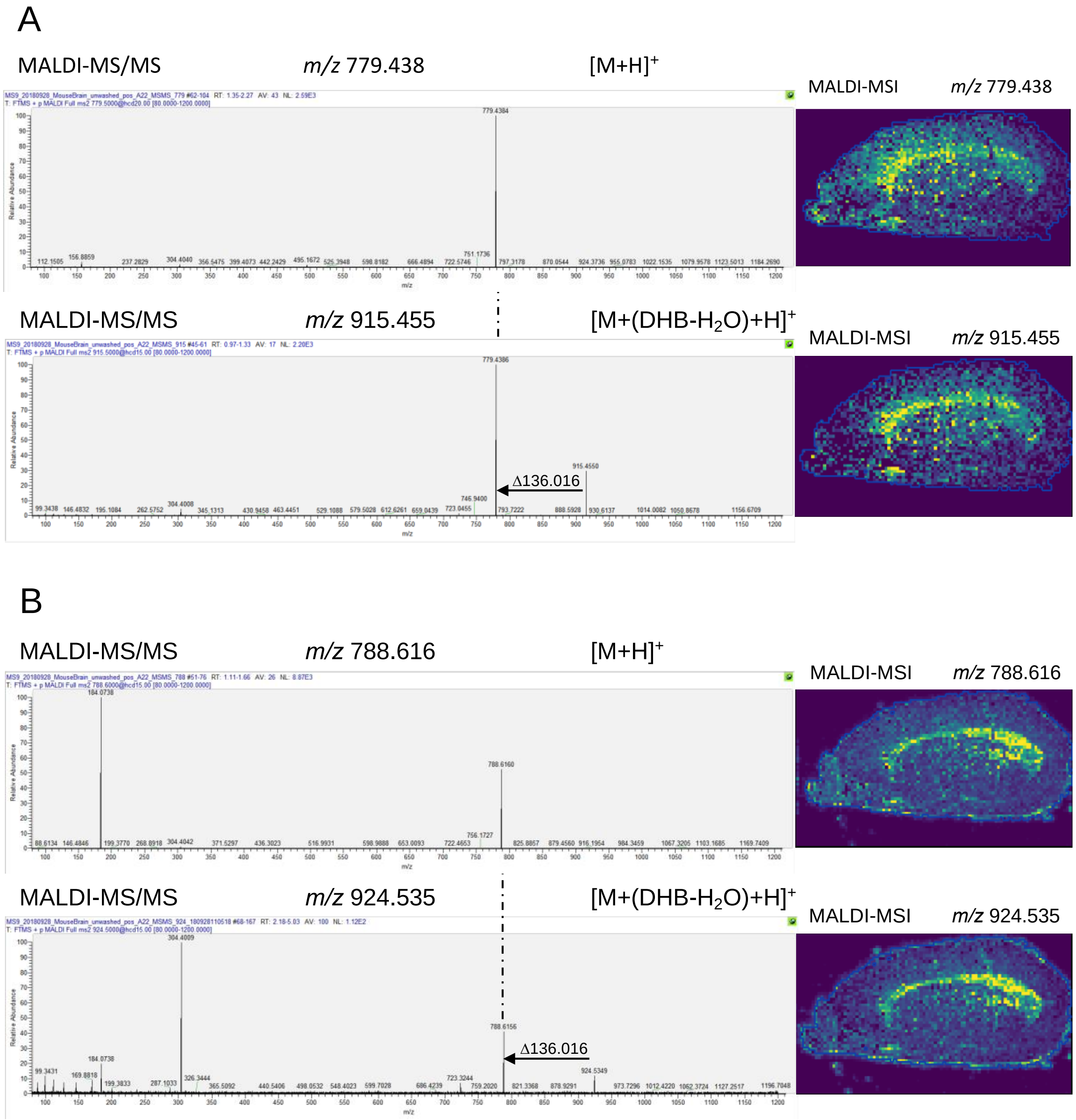

Figure S 2: MS^2 mass spectra and respective MS images of parent ions and DHB matrix adducts from mouse brain dataset #10. **A)** $[M+H]^+$ = *m/z* 779.438, $[M+(DHB\text{-}H_2O)+H]^+$ = *m/z* 915.455 **B)** $[M+H]^+$ = *m/z* 788.616, $[M+(DHB\text{-}H_2O)+H]^+$ = *m/z* 924.535

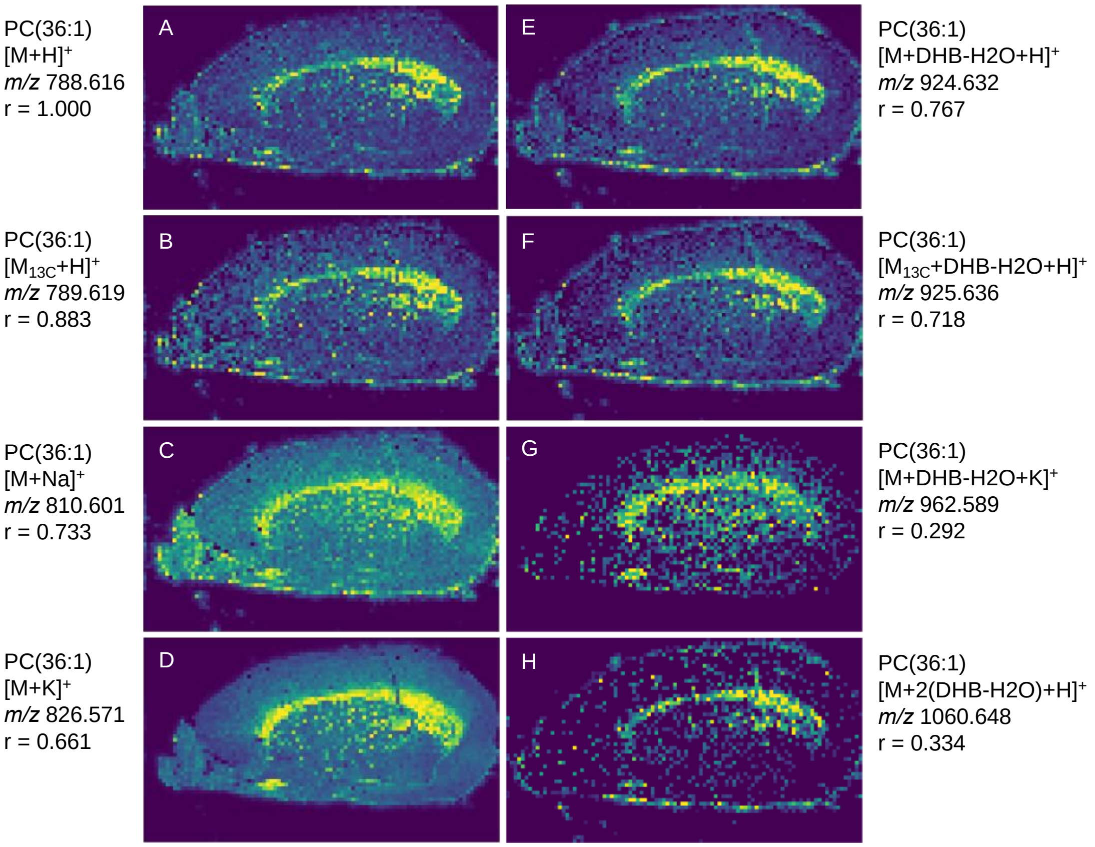

Figure S 3: Ion maps for PC(36:1) and respective adducts from mouse brain dataset #10. **A)** shows the most common ion $[M+H]^+$ as the parent ion. **B)-H)** show adducts of PC(36:1). Pearson correlation values of $[M+H]^+$ with respective adducts are given as r.

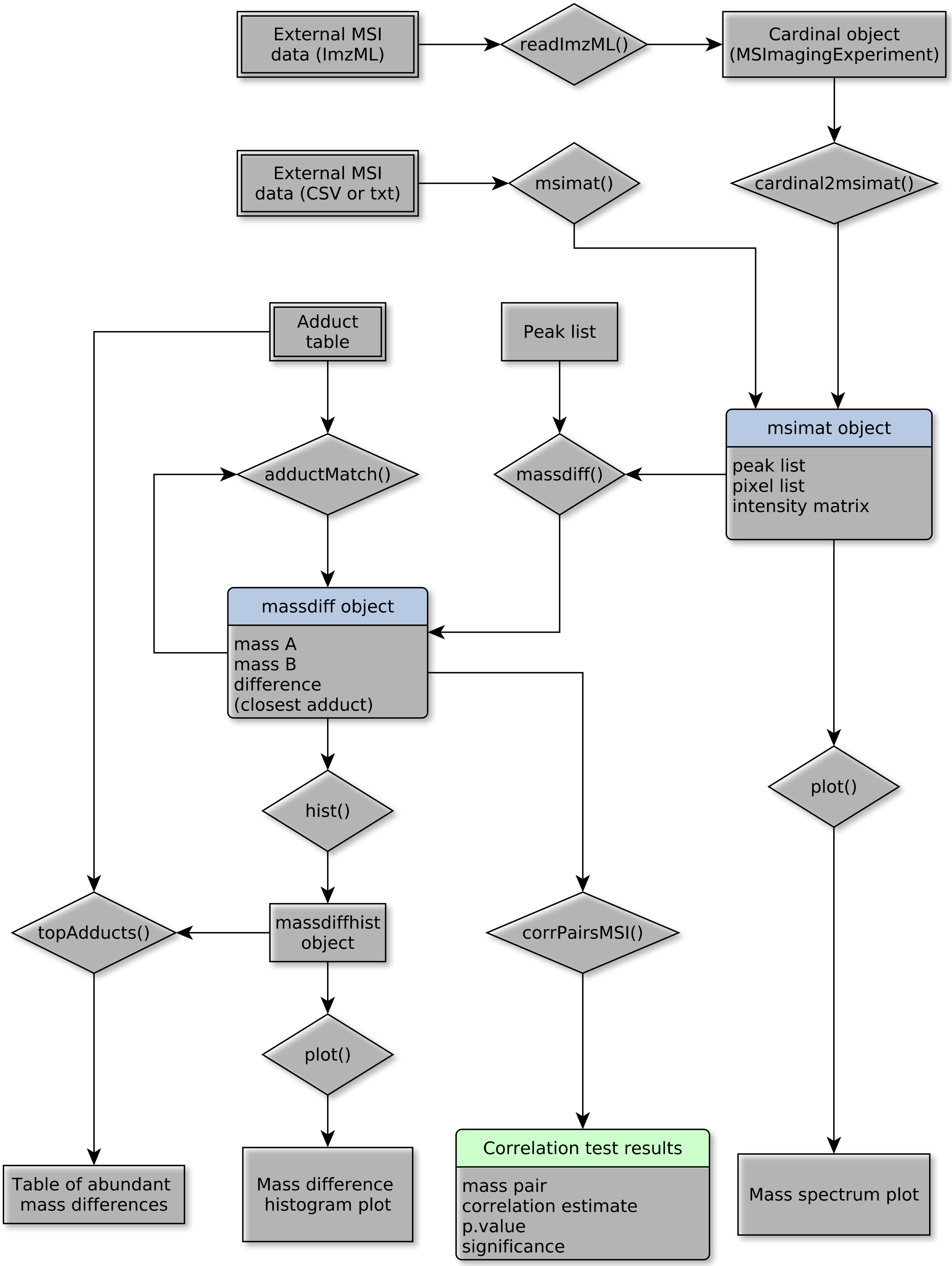

Figure S 4: *mass2adduct* workflow in R

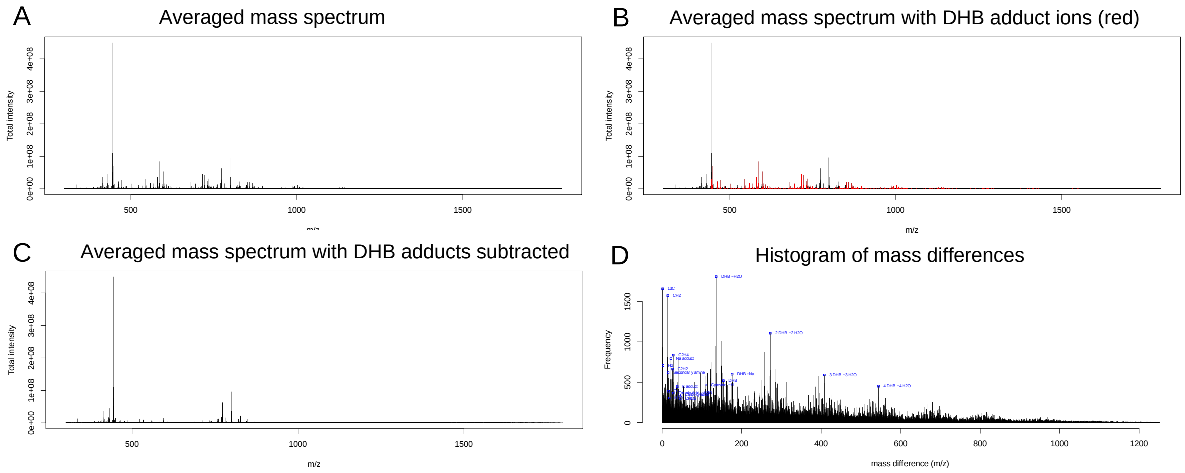

E

Top abundant mass differences

File	Mids	Counts	Density	Adduct name	Adduct formula	Adduct mass
10_Brain_Thermo	136,015	1810	0,097436085	DHB -H2O	C7H4O3	136,016
10_Brain_Thermo	1,005	1660	0,089361271	13C	13C	1,003
10_Brain_Thermo	14,015	1574	0,084731712	CH2	CH2	14,016
10_Brain_Thermo	272,035	1107	0,059592125	2 DHB -2 H2O	C14H8O6	272,032
10_Brain_Thermo	150,035	1008	0,054262748	NA	NA	NA
10_Brain_Thermo	135,015	947	0,05097899	NA	NA	NA
10_Brain_Thermo	0,005	932	0,050171509	NA	NA	NA
10_Brain_Thermo	258,015	873	0,046995416	NA	NA	NA
10_Brain_Thermo	28,035	834	0,044895964	C2H4	C2H4	28,031
10_Brain_Thermo	13,015	808	0,04349633	NA	NA	NA
10_Brain_Thermo	21,985	794	0,04274268	Na adduct	Na-H	21,982
10_Brain_Thermo	39,995	791	0,042581184	NA	NA	NA
10_Brain_Thermo	123,005	746	0,04015874	NA	NA	NA
10_Brain_Thermo	2,015	708	0,038113121	H2	H2	2,016
10_Brain_Thermo	149,025	704	0,037897792	NA	NA	NA
10_Brain_Thermo	27,025	680	0,036605822	NA	NA	NA
10_Brain_Thermo	26,015	662	0,035636844	C2H2	C2H2	26,016
10_Brain_Thermo	122,005	662	0,035636844	NA	NA	NA
10_Brain_Thermo	286,045	662	0,035636844	NA	NA	NA
10_Brain_Thermo	120,995	661	0,035583012	NA	NA	NA

F

Total ion map

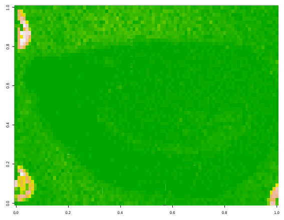

Figure S 5: Output options from *mass2adduct*. **A)** averaged mass spectrum **B)** averaged mass spectrum with DHB adduct ions (red) **C)** averaged mass spectrum with DHB adducts subtracted **D)** histogram of mass differences **E)** table of top abundant mass differences. Information on identity, formula and mass are added if mass differences match known adducts or chemical transformations. **F)** total ion map

Table S 1: List of mass differences used for data analysis. Formula for sodium, potassium and silver adducts are presented as substitution of a proton with the respective atom.

**Name**	**Formula**	**Mass**
**Adducts**		
Na adduct	Na -H	21.981945
K adduct	K -H	37.955885
^{107}Ag adduct	Ag -H	105.8973
NH4 adduct	NH3	17.0266
DHB adduct	C7H6O4	154.0266
DHB +Na adduct	C7H5O4Na	176.0086
DHB +K adduct	C7H5O4K	191.9825
DHB -H2O adduct	C7H4O3	136.016
DHB -H2O +Na adduct	C7H3O3Na	157.998
DHB -H2O +K adduct	C7H3O3K	173.972
2 DHB -2 H2O adduct	C14H8O6	272.032
3 DHB -3 H2O adduct	C21H12O9	408.048
4 DHB -4 H2O adduct	C28H16O12	544.064
5 DHB -5 H2O adduct	C35H20O15	680.08
CHCA adduct	C10H7NO3	189.0426
CHCA +Na adduct	C10H6NO3Na	211.0245
CHCA +K adduct	C10H6NO3K	226.9985
CHCA -H2O adduct	C10H5NO2	171.032
CHCA -H2O +Na adduct	C10H4NO2Na	193.014
CHCA -H2O +K adduct	C10H4NO2K	208.988
**Common chemical transformations**		
^{13}C Isotope	^{13}C -^{12}C	1.0033
Alanine -H2O	C3H5NO	71.03711384
Arginine -H2O	C6H12N4O	156.1011111
Asparagine -H2O	C4H6N2O2	114.0429275
Aspartic Acid -H2O	C4H5NO3	115.0269431
Cysteine -H2O	C3H5NOS	103.0091856
Cystine -H2O	C6H10N2O3S2	222.0132859
Glutamic Acid -H2O	C5H7NO3	129.0425932
Glutamine -H2O	C5H8N2O2	128.0585776
Glycine -H2O	C2H3NO	57.02146376
Histidine -H2O	C6H7N3O	137.0589119
(Iso)leucine -H2O	C6H11NO	113.0840641
Lysine -H2O	C6H12N2O	128.0949631
Methionine -H2O	C5H9NOS	131.0404858
Phenylalanine -H2O	C9H9NO	147.068414
Proline -H2O	C5H7NO	97.05276391
Serine -H2O	C3H5NO2	87.03202848
Threonine -H2O	C4H7NO2	101.0476785
Tryptophan -H2O	C11H10N2O	186.079313

Tyrosine -H2O	C9H9NO2	163.0633286
Valine -H2O	C5H9NO	99.06841398
Acetoacetic acid -H2O	C4H4O2	84.02112943
Acetone -H	C3H5O	57.03403983
Biotin -H	C10H15N2O3S	243.0803393
Biotin -H2O	C10H14N2O2S	226.0775996
Carbamoyl phosphate - PO4	CH2ON	44.01363872
Glutathione -H2O	C10H15N3O5S	289.0732426
Isoprene -H	C5H7	67.05477526
Malonic acid -H2O	C3H2O3	86.00039399
Pyridoxal phosphate -H2O	C8H8NO5P	229.0140109
Urea -H	CH3N2O	59.02453777
Adenine -H	C5H4N5	134.0466702
Adenosine -H2O	C10H11N5O3	249.0861894
Adenosine diphosphate -H2O	C10H13N5O9P2	409.0188541
Adenosine monophosphate -H2O	C10H12N5O6P	329.0525217
Cytidine diphosphate -H2O	C9H13N3O10P2	385.0076207
Cytidine monophsophate -H2O	C9H12N3O7P	305.0412884
Cytosine -H	C4H4N3O	110.0354368
Guanosine diphosphate -H2O	C10H13N5O10P2	425.0137687
Guanosine monophosphate -H2O	C10H12N5O7P	345.0474364
Guanine -H	C5H4N5O	150.0415848
Guanosine -H2O	C10H11N5O4	265.081104
Deoxythymidine diphosphate -H2O	C10H14N2O10P2	384.0123717
Thymidine -H2O	C10H12N2O4	224.079707
Thymine -H	C5H5N2O2	125.0351025
Thymidine monophosphate -H2O	C10H13N2O7P	304.0460394
Uridine diphosphate -H2O	C9H12N2O11P2	385.9916363
Uridine monophosphate -H2O	C9H11N2O8P	306.0253039
Uracil -H	C4H3N2O2	111.0194524
Uridine -H2O	C9H10N2O5	226.0589716
Acetic acid -H	C2H3O2	59.01330439
C2H2	C2H2	26.01565007
Carboxylation	CO2	43.98982928
CHO2	CHO2	44.99765432
H2O	H2O	18.01056471
C2H4	C2H4	28.03130015
Formic Acid -H2O	CO	27.99491464
Glyoxylate -H2O	C2O2	55.98982928
H2	H2	2.015650074
O	O	15.99491464
Phosphorus	P	30.9737634
Ketol group -H2O	C2H2O	42.01056471
CH2	CH2	14.01565007
Phosphoric acid -H2O	HPO3	79.966333

Primary amine	NH2	16.01872408
Diphosphoric acid -H2O	H2O6P2	159.932666
Secondary amine	NH	15.01089905
Sulfuric acid -H2O	SO3	79.95681572
Tertiary amine	N	14.00307401
C6H10O5	C6H10O5	162.0528236
C6H10O6	C6H10O6	178.0477382
Ribose -H2O	C5H8O4	132.0422589
Sucrose -H2O	C12H20O10	324.1056471
Glucose phosphate -H2O	C6H11O8P	242.0191559
Glucuronic acid -H2O	C6H8O6	176.0320881
Maltotriose -H2O	C18H30O15	486.1584707
Palmitic acid -H2O	C16H30O	238.2297
Palmitoleic acid -H2O	C16H28O	236.214
Stearic acid -H2O	C18H34O	266.261
Oleic acid -H2O	C18H32O	264.2453
Arachidic -H2O	C20H38O	294.2923
Eicosenoic acid -H2O	C20H36O	292.2766

Table S 2: Sample metadata

#	Dataset	Species	Matrix	Preprocessing	Reference
**1**	01_Mussel_DHB1	*Bathymodiolus puteoserpentis*	DHB	SCiLS	this study
**2**	02_Mussel_DHB2	*Bathymodiolus puteoserpentis*	DHB	SCiLS	this study
**3**	03_Mussel_DHB3	*Bathymodiolus puteoserpentis*	DHB	SCiLS	this study
**4**	04_Mussel_DHB4	*Bathymodiolus childressi*	DHB	Cardinal MSI	this study
**5**	05_Mussel_CHCA1	*Bathymodiolus puteoserpentis*	CHCA	SCiLS	this study
**6**	06_Mussel_CHCA2	*Bathymodiolus azoricus*	CHCA	SCiLS	this study
**7**	07_Mussel_CHCA3	*Bathymodiolus puteoserpentis*	CHCA	SCiLS	this study
**8**	08_Mussel_CHCA4	*Bathymodiolus childressi*	CHCA	Cardinal MSI	this study
**9**	09_Brain_Bruker_DHB	*Rattus*	DHB	SCiLS	upon request
**10**	10_Brain_Thermo_DHB	*Mus musculus*	DHB	Cardinal MSI	this study
**11**	11_Brain_MALDI_Waters_DHB	*Mus musculus*	DHB	HDImaging Water	https://science.sciencemag.org/content/348/6231/211
**12**	12_Brain_MALDI2_Waters_DHB	*Mus musculus*	DHB	HDImaging Water	https://science.sciencemag.org/content/348/6231/211
**13**	13_Testis_DHB	*Rattus*	DHB	SCiLS	https://s3-eu-west-1.amazonaws.com/testdata.scils.de/2019/rat_testis_Bruker_2019c.
**14**	14_Cocoa_DHB	*Theobroma cacao*	DHB	SCiLS	this study
**15**	15_Seagrass_DHB	*Posedonia*	DHB	SCiLS	this study
**16**	16_Fungus_DHB	*Claviceps purpurea*	DHB	SCiLS	http://link.springer.com/article/10.1007%2Fs00216-016-0047-2
**17**	17_MetaMix_DHB	Standards	DHB	Cardinal MSI	this study
**18**	18_MetaMix_CHCA	Standards	CHCA	Cardinal MSI	this study
**19**	19_Matrix_100_1000_DHB	DHB matrix control	DHB	SCiLS	this study
**20**	20_Matrix_500_2000_DHB	DHB matrix control	DHB	SCiLS	this study

Table S 3: Mass spectrometry metadata

#	m/z range	resolution	imgn col	ppm	peaks	MALDI source/ Detector	Mass resolution positive mode	RAW file name
**1**	350 - 1400	10 µm	355	2.777	3647	AP-SMALDI10 / Orbitrap	280000@200 m/z	20160914_DHB_pos_B.puteoserpentis_cryo_350-1400_10um_355x160_A31
**2**	475 - 1200	3 µm	500	4.115	1959	AP-SMALDI10 / Orbitrap	140000@200 m/z	20161014_Bathy_puteoserpentis_cryo_DHB_pos_FullMS_475-1200_3um_500x270_A35
**3**	500 - 2000	10 µm	315	9.41	1790	AP-SMALDI10 / Orbitrap	240000@200 m/z	MS9_20170210_DHB_pos_500-2000_10um_315x200_A28_B.put
**4**	80 - 900	10 µm	150	1	2002	AP-SMALDI10 / Orbitrap	240000@200 m/z	20181127_MS37_B_child_gill_80_900_DHB_pos_A28_10um_150x390
**5**	350 - 1400	10 µm	355	2	4002	AP-SMALDI10 / Orbitrap	280000@200 m/z	20160913_CHCA_pos_B.puteoserpentis_cryo_350-1400_10um_355x160_A31
**6**	500 - 1000	10 µm	100	2.554	3651	AP-SMALDI10 / Orbitrap	N/A	BAz3_100x320_10um_E20_1000ms_500-1000
**7**	450 - 1200	5 µm	275	6.048	1548	AP-SMALDI10 / Orbitrap	280000@200 m/z	20161125_CHCA_pos_Full450-1200_5um_275x250_A32_MALDI-
**8**	80 – 900	10 µm	155	1	609	AP-SMALDI10 / Orbitrap	240000@200 m/z	20181219_MS37_B_child_gill_80_900_HCCA_pos_A28_10um_155x420
**9**	200 - 1000	NA	0	2.825	1467	SmartBeam-II / MRMS	N/A	rat_brain_bruker_profile
**10**	300 - 1800	100 µm	96	1	1928	AP-SMALDI10 / Orbitrap	240000@200 m/z	MS9_20180924_MouseBrain_unwashed_300-1800_DHB_pos_A25_100um_96x55
**11**	50 - 2000	15 µm	0	5	4000	MALDI2 / QTOF	N/A	DHB_Subl_Kleinhirn_mit_LPI_Analyte_4_1
**12**	50 - 2000	15 µm	0	5	4000	MALDI2 / QTOF	N/A	DHB_Subl_Kleinhirn_mit_LPI_Analyte_5_1
**13**	150 - 3000	NA	0	11.036	5618	SmartBeam-II / MRMS	N/A	may2016_demo_scils_testis5600_10_profile
**14**	300 - 2000	100 µm	0	4.036	2390	AP-SMALDI10 / Orbitrap	280000@200 m/z	20160614_DHB_pos_300-2000_100um_154x246_A20_Cacaobean_72h
**15**	100 - 1000	12 µm	250	3.122	1860	AP-SMALDI10 / Orbitrap	240000@200 m/z	20161027_DHB_pos_Seagrass_root_FullMS_100-1000_12um_250x150_A30
**16**	60 - 2000	35 µm	0	5	4000	MALDI2 / QTOF	N/A	20140929_slide43_2_Analyte 1_1
**17**	60 – 900	100 µm	89	1	466	AP-SMALDI10 / Orbitrap	240000@200 m/z	20190123_MS37_MetaboliteMix_Spots_60_900_SDHB_pos_A28_100um_89x74
**18**	60 – 900	100 µm	75	1	376	AP-SMALDI10 / Orbitrap	240000@200 m/z	20190124_MS37_MetaboliteMix_Spots_60_900_HCCA_pos_A28_100um_75x89
**19**	100 - 1000	30 µm	32	5	N/A	AP-SMALDI10 / Orbitrap	240000@200 m/z	MS12_20170321_DHB_pos_100-1000_30um_32x32_A25_pure_matrix
**20**	500 – 2000	30 µm	32	5	N/A	AP-SMALDI10 / Orbitrap	240000@200 m/z	MS12_20170321_DHB_pos_500-2000_30um_32x32_A25_pure_matrix
